# Influence of Kiwifruit Extract Infusion on Consumer Sensory Outcomes of Striploin (*M. longissimus lumborum*) and Outside Flat (*M. biceps femoris*) from Beef Carcasses

**DOI:** 10.3390/foods8080332

**Published:** 2019-08-08

**Authors:** Angela Lees, Małgorzata Konarska, Garth Tarr, Rod Polkinghorne, Peter McGilchrist

**Affiliations:** 1 School of Environmental and Rural Science, University of New England, Armidale 2350, Australia; 2 Division of Engineering in Nutrition, Warsaw University of Life Sciences, 02-787 Warsaw, Poland; 3 School of Mathematics and Statistics, The University of Sydney, Sydney 2006, Australia; 4 Birkenwood Pty. Ltd., Murrurundi 2338, Australia; 5 School of Veterinary & Life Sciences, Murdoch University, Perth 6150, Australia

**Keywords:** actinidin, consumer acceptance, consumer sensory testing, eating quality, grass-fed beef, Meat Standards Australia (MSA), proteolysis, sensory testing

## Abstract

Actinidin is a cysteine protease enzyme which occurs in kiwifruit and has been associated with improved tenderness in red meat. This study evaluated the impact of actinidin, derived from kiwifruit, on consumer sensory outcomes for striploin (*M. longissimus lumborum*) and outside flat (*M. biceps femoris*). Striploins and outside flats were collected from 87 grass-fed steers. Carcasses were graded to the Meat Standards Australia (MSA) protocols. Striploins and outside flats were then dissected in half and allocated to one of the following two treatments: (1) not infused (control) and (2) infused with a kiwifruit extract (enhanced), and then prepared as grill and roast samples. Grill and roast samples were then aged for 10 or 28 days. Consumer evaluations for tenderness, juiciness, flavor, and overall liking were conducted using untrained consumer sensory panels consisting of 2080 individual consumers, in accordance with the MSA protocols. These scores were then used to calculate an overall eating quality (MQ4) score. Consumer sensory scores for tenderness, juiciness, flavor, overall liking, and MQ4 score were analyzed using a linear mixed-effects model. Kiwifruit extract improved consumer scores for tenderness, juiciness, flavor, overall liking, and MQ4 scores for striploins and outside flat (*p* < 0.05). These results suggest that kiwifruit extract provides an opportunity to improve eating experiences for consumers.

## 1. Introduction

A challenge to the beef industry has been to provide consumers with a consistent and enjoyable eating experience. Lyford et al. [1] established that consumers are willing to pay higher premiums for higher quality beef. In Australia, the eating quality of beef is underpinned by the Meat Standards Australia (MSA) cut-based grading program [2]. The development of eating quality predictors was conducted through numerous consumer sensory panels evaluating the tenderness, juiciness, flavor, and overall liking of 39 cuts from beef carcasses. These cuts can be prepared using up to eight different methods [3], providing eating quality predictions for 135 cut × cook method combinations [2]. Traditionally, eating quality predictions have been used to assign one of four quality grades consisting of: (1) unsatisfactory, (2) “3 star” good everyday quality, (3) “4 star” better than everyday quality, and (4) “5 star” premium quality [4,5]. However, it is well established that eating quality is highly variable across different cuts and cut × cook method combinations [6]. Eating quality is impacted by numerous animal and management factors including, but not limited to the following: *Bos indicus* content, carcass hanging method, intramuscular fat, ultimate pH (pHu), and post-mortem aging of the cut and cooking method [5]. Thus, eating quality of beef can be influenced by many facets of the supply chain [2,4]. However, to ensure consumer enjoyment and satisfaction of all beef purchases, improving the potential consumer experience of unsatisfactory and lower quality cuts requires value adding, which also has the potential to increase industry profitability.

Techniques for value adding, preparing, and cooking beef are continually growing, increasing the value proposition of all muscles in the carcass. Kiwifruit (*Actinidia deliciosa*) contains a cysteine protease enzyme, actinidin [7], and has been identified to improve the tenderness in beef [8,9,10], lamb [11] and pork [12]. Toohey et al. [8] concluded that kiwifruit infusion of beef topsides (*M. semimembranosus*) reduced shear force values. The findings of Toohey et al. [8] suggest that kiwifruit extract infusions may improve consumer tenderness scores. Therefore, a kiwifruit extract infusion has the potential to increase the quality grades of low-quality cuts, improving the potential value of the cut, while improving consumer satisfaction. Eating quality, as defined by the MSA model, evaluates consumer satisfaction by four attributes including: (1) tenderness, (2) juiciness, (3) flavor, and (4) overall liking. These attributes are then used to determine an overall eating quality score (MQ4) for each cut × cook method combination. The influence of actinidin derived from kiwifruit on consumer sensory outcomes has not yet been elucidated for any cook method. Therefore, the hypothesis of this study was that consumer sensory scores will improve for the striploin (*M. longissimus lumborum*) and outside flat (*M. biceps femoris*) after infusion with a kiwifruit extract solution cooked as a grill or roast.

## 2. Materials and Methods

### 2.1. Animals

A total of 87 grass-fed steers were utilized in the current study. All cattle were sourced from a single cross-breeding herd comprising of *Bos indicus* crossbred maternal lines and were sired by Red Poll, Wagyu, and Brahman bulls. Hormone growth promotants (HGPs) were not used within this study.

### 2.2. Slaughter Procedure, Carcass Grading, and Muscle Collection

After 8 h in lairage, cattle were slaughtered at a commercial abattoir (Queensland, Australia). Post-slaughter carcasses were marshalled into a spray chiller. Carcass temperature and pH rate of decline were recorded at hourly intervals from chiller entry until a muscle pH of 6 was obtained [2,5]. This was done to ensure that all carcasses passed through pH 6 between 15 °C and 35 °C, ensuring conformance to MSA pH and temperature decline requirements [5,13]. Post chilling, carcasses were evaluated by a single accredited MSA grader 20 h post slaughter [13]. Hump heights were measured during carcass grading using a 5 mm graduated metal ruler [6]. Striploins (*M. Longissimus lumborum*) and outside flats (*M. biceps femoris*) from the left side of each carcass were collected at boning. Striploins and outside flats were vacuum packed and chilled for 24 h prior to collection from the abattoir. These muscles were then transported by refrigerated transport at 1 °C for further processing.

### 2.3. Muscle Preparation

On day 6, striploins and outside flats were dissected from any secondary muscles and denuded to remove all external fat and epimysium. Striploins and outside flats were then dissected in half and then allocated to one of two treatments: (1) not infused (control) and (2) infused with a kiwifruit extract (enhanced). The kiwifruit infusion solution was prepared according to the specifications, where 10 kg kiwifruit extract was completely dissolved in 72 L of water (Earlee Products Pty Ltd.; Wunda Brine CFD 5000, Code: 044-224M, Batch No: 170727, Brisbane, Australia). Enhanced muscle portions were injected at a rate of approximately 10% initial weight using a Fomaco Machine equipped with 4 mm needles (Copenhagen, Denmark). Post-enhancement muscle samples were reweighed to determine change in muscle weights associated with the enhancement process.

Enhanced and control striploins and outside flat samples were then prepared as grill (GRL) and roast (RST) samples, as per the MSA protocols as described by Watson et al. [14,15]. Briefly, GRL and RST samples were portioned into 75 × 25 × 150 mm and 75 × 75 × 150 mm portions, respectively. Grill samples were then individually wrapped in freezer film prior to packing into vacuum sealed bags [15]. Roast samples were individually packed into labelled vacuum-sealed bags [15]. Samples were then aged at 1 °C until 10 or 28 days after slaughter (4 and 22 days post-infusion treatment) at which point they were frozen and stored at −20 °C until being thawed for consumer sensory testing.

### 2.4. Consumer Sensory Testing

Consumer sensory testing sessions were conducted using the MSA protocols, as described by Watson et al. [14,15]. Briefly, GRL samples were cooked on a Silex grill (Silex S-Tronic Single Grill, Piotis Pty Ltd., Marrickville, Australia) heated to approximately 200 °C. Grill samples were cooked on the Silex in a predetermined order for 5 min to ensure samples were cooked to a medium doneness, samples were then rested for 2 min as per the MSA protocols, as described by Watson et al. [14,15]. Samples were then halved and served to two consumers.

Roast samples were presented to untrained consumers in three presentations (i) as a hot roast (RST) sample as per standard MSA protocol, portioned into a 10 mm slice [15]; (ii) cold roast sample portioned as a 10 mm slice (RST_10_); and then (iii) cold roast sample portioned as a 2 mm slice (RST_2_). Roast samples were cooked in a commercial fan-forced gas oven at 160 °C until samples reached an internal temperature of 65 °C. Samples were stored in a Bain Marie for a minimum of 5 min until preparation for serving. Samples were then trimmed to a standard size of 65 × 65 × 110 mm, before being returned to a Bain Marie maintained at 48 °C. Prior to service, the RST samples were portioned into 10 mm slices. The remaining roast samples were placed in a specifically designed roast holder and chilled overnight, and then served cold to a different consumer group (the following day). This was identical to the hot service, where RST_10_ samples were prepared into 10 mm slices and offered to a second group of consumers. Similarly, at the conclusion of this service the final remaining section of the roast was prepared as a RST_2_ on a deli slicer, set at 2 mm, then served to a third group of consumers, two days after the initial hot roast sensory testing. Excluding these exceptions, other procedures followed the MSA protocols as described by Watson et al. [14,15].

Grill and RST samples were presented in a controlled 6 × 6 latin square design ensuring that each sample was presented an equal number of times in the serving order from two to seven and an equal number of times before and after each of the other products, effectively balancing for potential order or halo effects [15]. Samples were prepared over 35 consumer sensory sessions consisting of 60 untrained consumers in each session, suggesting 2100 consumer scores. However, there were 20 untrained consumers that did not consume all sensory samples, thus a total of 2080 individual consumers were used within the current study. Consumers (*n* = 2080) evaluated each sample served for tenderness, juiciness, flavor, and overall liking by scoring a line on a 100 mm visual analogue scale ranging from 0–100. The visual analogue scale was anchored by descriptions which were not tender/very tender, not juicy/very juicy, and dislike extremely/like extremely for both liking of flavor and overall liking scores, i.e., 0 indicated a not tender sample and 100 was used to describe a very tender sample.

### 2.5. Statistical Analysis

The overall eating quality score (MQ4) was determined from the consumer tenderness, juiciness, flavor, and overall liking [6]. Tenderness, juiciness, flavor, and overall liking scores were weighted by 0.4, 0.1, 0.2, and 0.3, respectively, providing a MQ4 score between 0 and 100 [6]. The raw means of each sensory trait were calculated together with clipped means calculated by removing the highest and lowest 2 scores for each trait [6].

All data exploration and statistical analyses were conducted in R [16]. Data merging and manipulation, data visualizations, and summary data were conducted using the “dplyr” [17], “ggplot2” [18], and “table1” [19] packages, respectively.

Initially, correlations between raw and clipped consumer sensory scores for meat tenderness, juiciness, flavor, overall liking, and MQ4 score were conducted. Raw and clipped consumer sensory scores for meat tenderness, juiciness, flavor, overall liking, and MQ4 score were analyzed using a linear mixed-effects model in the “lme4” package [20] and estimated marginal means were generated using the “emmeans” package [21].

Models incorporated muscle, number of days aged, treatment and cooking method, and their interactions as fixed effects. Models were refined to remove relevant insignificant interactions in a step-wise manner. The final models for tenderness, juiciness, flavor, overall liking, and MQ4 included muscle, number of days aged, treatment, cooking method, muscle × treatment, number of days aged × treatment, muscle × treatment, muscle × cooking method, number of days aged × cooking method, treatment × cooking method, and number of days aged × treatment × cooking method. Additionally, an individual animal/carcass identification was incorporated as a random effect in all models, to account for animal factors. The term cooking method was used to describe GRL, RST, RST_2_, and RST_10_.

## 3. Results

Strong correlations between raw and clipped consumer sensory scores for tenderness (R^2^ = 0.99, *p* ≤ 0.0001), juiciness (R^2^ = 0.99, *p* ≤ 0.0001), flavor (R^2^ = 0.99, *p* ≤ 0.0001), overall liking (R^2^ = 0.99, *p* ≤ 0.0001) and MQ4 (R^2^ = 0.99, *p* ≤ 0.0001) were identified. Furthermore, there were no differences in the significant terms between models conducted on raw and clipped data, therefore, data herein pertains to analysis conducted on the raw consumer sensory data.

### 3.1. Carcass Traits

All 87 carcasses were graded as per the MSA grading specifications [13]. Carcass characteristics as evaluated by the MSA carcass grading specifications are summarized in Table 1.

### 3.2. Kiwifruit Extract Infusion

The average weight increase post kiwifruit extract enhancement was 11.2% ± 0.29%, however, there was some variability in the enhancement rate between striploin and outside flat muscles (Table 2).

### 3.3. Consumer Sensory Outcomes

Overall, on average striploins had greater tenderness, juiciness, flavor, overall liking, and MQ4 scores as compared with outside flats (*p* < 0.001, Table 3). Consumers exhibited an increased preference for RST_2_ samples (*p* < 0.001, Table 4).

The number of days muscles were aged did not influence consumer evaluations of tenderness (*p* = 0.11), juiciness (*p* = 0.59), flavor (*p* = 0.2), overall liking (*p* = 0.7) or MQ4 (*p* = 0.99) scores. Furthermore, there were no number of days aged × cooking method influences on consumer perceptions of tenderness (*p* = 0.65), juiciness (*p* = 0.23), flavor (*p* = 0.55), overall liking (*p* = 0.7) or MQ4 (*p* = 0.67) scores.

**Figure 1:**
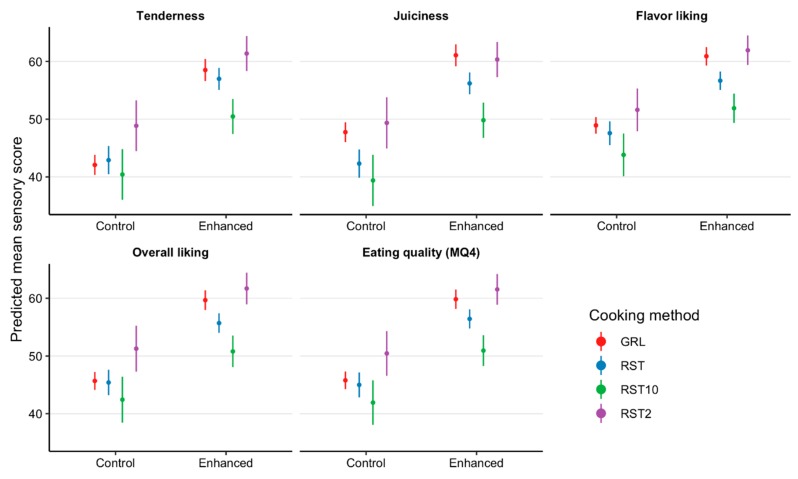
Estimated marginal means with 95% confidence intervals for tenderness, juiciness, flavor, overall liking, and eating quality (MQ4) scores of striploins (*M. longissimus lumborum*) and outside flats (*M. biceps femoris*) not infused (control) and infused with a kiwifruit extract (enhanced) presented to consumers (*n* = 2080) as grill (GRL), 10 mm hot roast (RST), 2 mm cold roast (RST_2_) and 10 mm cold roast (RST_10_).

Kiwifruit extract infusion improved consumer evaluations of tenderness, juiciness, flavor, overall liking, and MQ4 by 11.4, 13.2, 12.2, 9.9, and 10.8 points, respectively, (*p* < 0.001), when compared to the control samples. Similarly, there was a treatment × cooking method interaction as the GRL, RST, RST_2_, and RST_10_ tenderness scores increased by 16.4, 14.1, 10.1, and 12.5 points (*p* = 0.04, Figure 1); overall liking scores increased by 14, 10.3, 8.4, and 10.4 points (*p* = 0.01, Figure 1); and MQ4 scores increased by 14, 11.4, 9, and 11.1 points (*p* = 0.05, Figure 1), respectively, when compared with the control samples. However, there was no effect on consumer juiciness (*p* = 0.36, Figure 1) or flavor (*p* = 0.08, Figure 1) scores.

**Figure 2:**
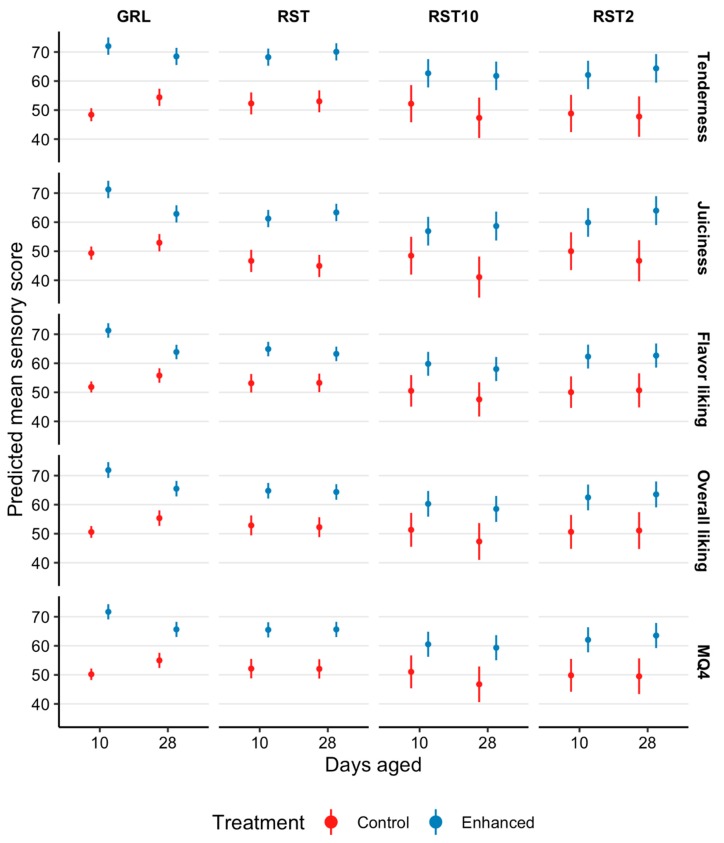
Estimated marginal means with 95% confidence intervals for tenderness, juiciness, flavor liking, overall liking, and eating quality (MQ4) scores of striploins (STR045; *M. longissimus lumborum*) presented to consumers (*n* = 2080) as grill (GRL), 10 mm hot roast (RST), 2 mm cold roast (RST_2_), and 10 mm cold roast (RST_10_) that were not infused (control) and infused with a kiwifruit extract (enhanced), and aged for 10 or 28 days.

**Figure 3:**
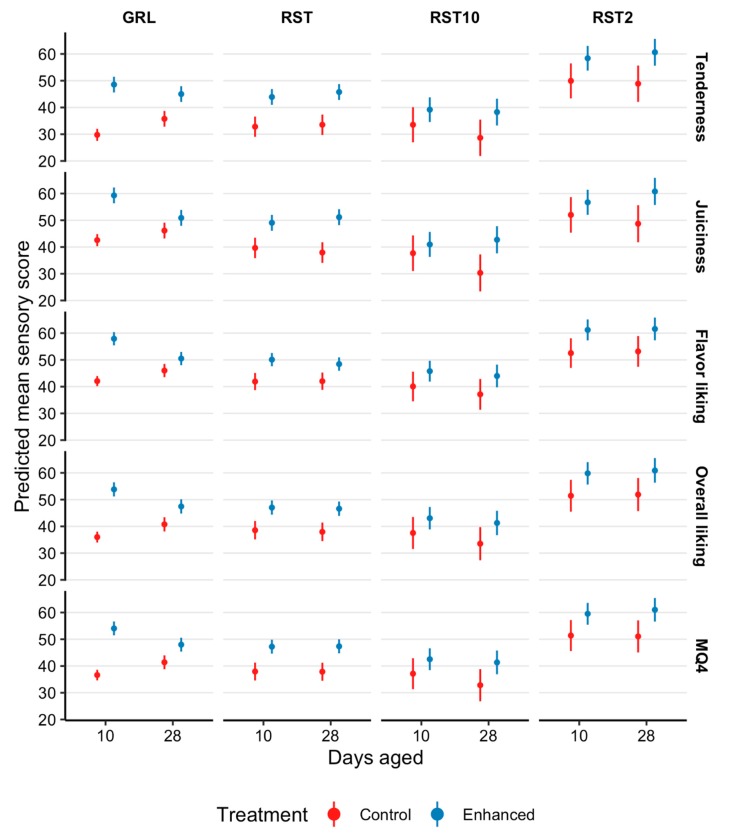
Estimated marginal means with 95% confidence intervals for tenderness, juiciness, flavor liking, overall liking, and eating quality (MQ4) scores of outside flats (OUT005, *M. biceps femoris*) presented to consumers (*n* = 2080) as grill (GRL), 10 mm hot roast (RST), 2 mm cold roast (RST_2_) and 10 mm cold roast (RST_10_) that were not infused (control) and infused with a kiwifruit extract (enhanced), and aged for 10 or 28 days.

**Figure 4:**
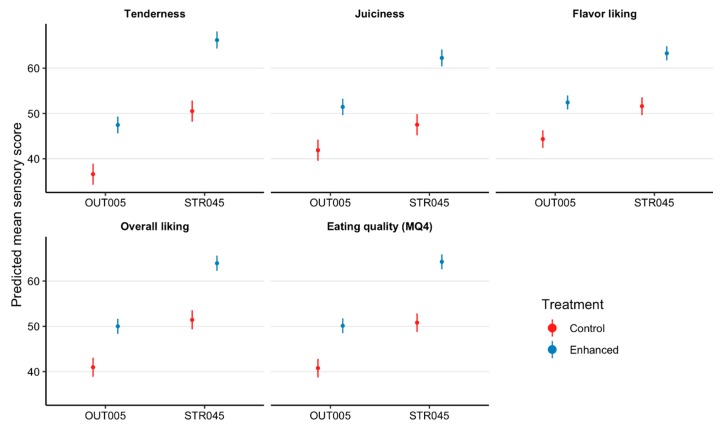
Estimated marginal means with 95% confidence intervals for tenderness, juiciness, flavor, overall liking, and eating quality (MQ4) scores of striploins (STR045, *M. longissimus lumborum*) and outside flats (OUT005, *M. biceps femoris*) presented to consumers (*n* = 2080) that were not infused (control) and infused with a kiwifruit extract (enhanced).

Kiwifruit infusion improved consumer scores for tenderness, juiciness, flavor, overall liking, and MQ4 of striploins (Figure 2) by between 11.7 and 15.7 points and outside flats (Figure 3) by between 8 and 10.9 points (*p* < 0.02; Figure 2, Figure 3 and Figure 4), relative to the control samples. Furthermore, there was a number of days aged × treatment interactions with consumer perceptions of tenderness (*p* = 0.02), juiciness (*p* = 0.05), flavor (*p* = 0.0001), overall liking (*p* = 0.0006), and MQ4 (*p* = 0.0009), however, there was a reduction in consumer acceptance of the enhanced treatment at 28 days aging (Figure 2 and Figure 3).

There were a number of days aged × treatment × cooking method influences (Figure 2 and Figure 3) on consumer evaluations of tenderness (*p* = 0.005), juiciness (*p* < 0.001), flavor (*p* = 0.003), overall liking (*p* = 0.001), and MQ4 (*p* = 0.0007). However, consumer tenderness evaluations had similar increases for RST, RST_2_, and RST_10_ samples aged for 10 and 28 days. At 10 days aging there was an increase of 13.6, 10.8, and 8 points for enhanced RST, RST_2_, and RST_10_ as compared with the control samples. Comparably, at 28 days aging, consumer evaluations for tenderness increased 14.6, 14.2, and 12 pointsfor the enhanced RST, RST_2_ and RST_10_.

Consumer sensory scores indicated that the juiciness of enhanced GRL samples that were aged for 10 days, were 19.3 points higher than control samples. Whereas juiciness scores for enhanced GRL that were aged for 28 days, increased by 7.4 points. The kiwifruit extract increased the juiciness scores of RST, RST_2_, and RST_10_ that were aged for 10 days by 11.9, 7.2, and 5.8 points, respectively, when compared with the control samples. Although the consumer evaluations of juiciness exhibited an increase of 15.9, 14.7, and 12 points in tenderness scores of enhanced RST, RST_2_, and RST_10_ that were aged for 28 days, relative to the control samples.

Flavor scores highlight that enhanced GRL samples that were aged for 10 days, were 17.6 points higher than control samples, however, 28 days aging only observed an increase of 6.3 points. Similar increases in consumer scores were observed for enhanced RST (10 days, 10 points and 28 days, 8.2 points), RST_2_ (10 days, 10.5 points and 28 days, 10.2 points), and RST_10_ (10 days, 7.5 points and 28 days, 8.7 points) samples aged for 10 and 28 days.

At 10 days aging, there was an increase of 19.6 points in consumer overall liking for enhanced GRL samples, relative to the control samples. Although, increasing the number of days aged to 28 days only increased the overall liking of enhanced GRL samples by 8.4 points. Consumer overall liking scores had similar increases for enhanced RST (10 days, 10.2 points and 28 days, 10.4 points), RST_2_ (10 days, 10.2 points and 28 days, 10.7 points) and RST_10_ (10 days, 7.3 points and 28 days, 9.5 points) samples aged for 10 and 28 days, when compared with the control samples.

There was an increase of 19.5 points in MQ4 scores of enhanced GRL samples aged for 10 days, relative to the control samples. However, this did not persist to 28 days aging where the increase in MQ4 was 8.6 points. Additionally, there were increases in MQ4 scores of enhanced RST (10 days, 11.4 points and 28 days, 11.5 points), RST_2_ (10 days, 10.2 points and 28 days, 12 points) and RST_10_ (10 days, 7.4 points and 28 days, 10.5 points) samples aged for 10 and 28 days, in comparison to the control samples.

## 4. Discussion

This study is the first to evaluate the influence of infusing beef cuts with actinidin derived from kiwifruit on untrained consumer sensory panels. The results from this study show that infusing striploins and outside flats with a kiwifruit extract improved consumer scores for tenderness, juiciness, flavor, overall liking, and MQ4 scores, which supported the initial hypothesis. Specifically, there was an increase of 13.4 points in the MQ4 score of enhanced striploins, which related to a quality grade increase from a 3-star good everyday product to a 4-star better than everyday quality product [4,5]. In comparison, outside flats had an increase of 9.3 points in the MQ4 score, which indicated a quality grade increase from unsatisfactory to a 3-star good everyday product [4,5]. Therefore, it is clearly evident that the kiwifruit enhancement was capable of improving consumer acceptance of beef striploins and outside flats, providing an opportunity for the beef industry to generate greater revenue from these primals.

Similarly, for GRL, RST, RST_2_, and RST_10_ infusion with kiwifruit extract increased the MQ4 scores from 45.8 ± 0.78, 45.0 ± 1.10, 50.4 ± 1.97, and 41.9 ± 1.97 to 59.8 ± 0.85, 56.4 ± 0.85, 61.5 ± 1.36, and 50.9 ± 1.36. This equates to an increase in the eating quality predictions of RST and RST_10_ from an unsatisfactory product to a 3-star good everyday grade [4,5]. However, for GRL and RST_2_, kiwifruit extract improved the eating quality from a low grade 3-star good everyday quality to a higher good everyday quality [4,5]. This suggests that the kiwifruit extract improved the overall eating experience of consumers while not impacting on the flavor of striploins or outside flats. Similarly, Christensen et al. [12] highlighted similar findings in pork *M. biceps femoris*. The authors reported that an improvement in textural attributes, while juiciness, flavor, and taste were not influenced by actinidin infusions [12]. Overall, this suggests that kiwifruit extract can be used to effectively tenderize meat without adversely affecting other sensory attributes.

At 10 and 28 days aging, enhanced GRL, RST, RST_2_ and RST_10_ exhibited increases in consumer evaluations of tenderness, juiciness, flavor, overall liking, and MQ4 scores, as compared to the control samples. However, tenderness, juiciness, flavor, overall liking, and MQ4 scores for enhanced samples aged for 28 days generally decreased in comparison to enhanced samples that were aged for 10 days, or four days post infusion. Regardless, beef infused with the kiwifruit solution and aged for 28 days still had higher consumer sensory scores as compared with the control samples. The mechanisms that resulted in the decreased consumer scores for enhanced samples aged for 28 days as compared with 10 days are yet to be clarified.

Lewis and Luh [10] indicated that actinidin may hydrolyze muscle proteins that are associated with toughness in beef cuts, rather than having a hydrolyzing action on all of the proteins in beef muscle. The tenderization of beef is a complex process that involves numerous structural changes in myofibrillar components associated with proteolytic enzymes [9]. This suggests that the improvement of eating quality from kiwifruit extracts may be due to an increased proteolytic activity on muscle proteins [11], possibly specifically myofibrillar proteins [8], by actinidin.

The results presented here suggest that proteolysis is sustained in beef cuts infused with kiwifruit extracts up to 28 days and possibly longer. This suggests that there may be a risk of over tenderization occurring due to the continued proteolytic activity [22]. Hence, aging beyond 10, or 14 days [8], may not provide the optimum eating experience for consumers. As such, these results suggest that the optimum number of days for aging beef infused with kiwifruit extracts warrants further investigations. Furthermore, it is important to consider that the optimum number of days for aging may vary across different cuts of meat, as some beef cuts may benefit from a longer period of aging [14,23]. Understanding the optimum number of days for aging post infusion process would ensure that a consistent and enjoyable eating experience for consumers is achieved.

This study has highlighted that using a kiwifruit infusion improved the quality grades of striploin and outside flats within this study, suggesting that kiwifruit extract provides an opportunity to improve the value of low- and poor-quality beef. Lyford et al. [1] indicated that consumer willingness to pay increased by approximately 50% for increased quality of (1) unsatisfactory to 3-star good everyday quality and (2) 3-star good to 4-star better everyday quality beef. Therefore, developing value adding methods for improving the quality grades of low-quality cuts is important for the long-term financial sustainability of the beef industry. On the basis of these findings, it could be reasoned that the kiwifruit infusion of low-quality striploins and outside flats has the potential to ”double” the market value within Australia. Nevertheless, these results suggest that cuts infused with the kiwifruit solution should be cooked or frozen within a short period after the infusion process is conducted to reduce the activity of actinidin and ensure an optimum consumer experience.

## 5. Conclusions

Enhancing striploins and outside flats improved the eating quality scores of GRL, RST, RST_2_, and RST_10_ as evaluated by untrained consumers. In both muscles, at 10 and 28 days post-mortem aging, consumed as GRL or RST, the tenderness, juiciness, flavor, and overall liking of the kiwifruit enhanced product was increased, improving the intrinsic eating quality. As such, this study suggests that kiwifruit extract has the potential to increase consumer acceptance while increasing profitability of processors by improving unsatisfactory and low-quality beef cuts into acceptable and high-quality cuts.

## Figures and Tables

**Table 1:** Mean (± SEM), minimum and maximum carcass characteristics as determined by the Meat Standards Australia (MSA) beef carcass grading specifications.

Carcass Trait	Mean	Minimum	Maximum
HSCW, kg	301.6 ± 2.1	262	357.5
Hump height, mm	86.2 ± 2.4	40	150
Eye muscle area, cm^2^	79.3 ± 0.94	65	100
Rib fat, mm	7.5 ± 0.32	3	19
Ossification	155.4 ± 2.1	130	230
MSA marbling score	327.5 ± 7.8	220	510
pHu	5.5 ± 0.01	5.43	6.12

**Table 2:** Mean (± SEM), minimum, and maximum increase in muscle weights for striploins (*M.* *longissimus lumborum*) and outside flats (*M. biceps femoris*) enhanced with a kiwifruit extract.

Item	Mean	Minimum	Maximum
Striploin			
Initial weight, kg	2.18 ± 0.04	1.39	3.2
Final weight, kg	2.41 ± 0.05	1.54	3.5
Percent increase, %	10.75 ± 0.04	4.54	16.2
Outside flat			
Initial weight, kg	1.41 ± 0.03	0.82	2.31
Final weight, kg	1.57 ± 0.03	0.87	2.56
Percent increase, %	11.64 ± 0.35	5.83	25

**Table 3:** Estimated marginal means (± 95% confidence interval) and the difference between striploins (*M.* *longissimus lumborum*) and outside flats (*M. biceps femoris*) scores for tenderness, juiciness, flavor, overall liking, and eating quality (MQ4) ^1,2^.

Consumer Scores	Striploin	Outside Flat	Difference
Tenderness	58.4 ± 0.86 ^a^	42 ± 0.86 ^b^	16.4
Juiciness	54.9 ± 0.84 ^a^	46.7 ± 0.84 ^b^	8.2
Flavor	57.4 ± 0.72 ^a^	48.4 ± 0.71 ^b^	9
Overall liking	57.7 ± 0.76 ^a^	45.5 ± 0.76 ^b^	12.2
MQ4	57.5 ± 0.76 ^a^	45.4 ± 0.75 ^b^	12.1

^1^ Tenderness, juiciness, flavor, and overall liking were scored by consumers on a 100 mm visual analogue scale ranging from 0 to 100. ^2^ Eating quality scores (MQ4) were calculated by weighting tenderness (0.4), juiciness (0.1), flavor (0.2), and overall liking (0.3) providing a MQ4 score between 0 and 100 [6]. ^3^ Data presented represents consumer sensory scores from 2080 individual untrained consumers. ^a,b^ Within a row, means without a common superscript differ (*p* < 0.0001).

**Table 4:** Estimated marginal means (± 95% confidence interval), and difference between striploins (*M.* *longissimus lumborum*) and outside flats (*M. biceps femoris*) scores for tenderness, juiciness, flavor, overall liking, and eating quality (MQ4) served as grill (GRL), hot roast (RST), cold roast as a 2 mm slice (RST_2_) and cold roast as a 10 mm slice (RST_10_) for tenderness, juiciness, flavor, overall liking, and eating quality (MQ4) ^1,2,3^.

Consumer Scores	Striploin				Outside Flat				Difference ^4^			
	GRL	RST	RST_2_	RST_10_	GRL	RST	RST_2_	RST_10_	GRL	RST	RST_2_	RST_10_
Tenderness	60.8 ± 0.93	60.9 ± 1.11	55.8 ± 1.89	56 ± 1.89	39.8 ± 0.91	39 ± 1.11	54.5 ± 1.87	34.9 ± 1.87	21 *	21.9 *	1.3	21.1 *
Juiciness	59.1 ± 0.91	54 ± 1.1	55.1 ± 1.91	51.3 ± 1.91	49.7 ± 0.9	44.5 ± 1.11	54.6 ± 1.88	37.9 ± 1.88	9.4 *	9.5 *	0.5	13.4 *
Flavor	60.7 ± 0.77	58.6 ± 0.93	56.4 ± 1.6	54 ± 1.6	49.1 ± 0.76	45.6 ± 0.93	57.1 ± 1.57	41.7 ± 1.57	11.6 *	13 *	−0.7	12.3 *
Overall liking	60.8 ± 0.82	58.6 ± 1	56.9 ± 1.72	54.4 ± 1.72	44.5 ± 0.81	42.5 ± 1	56 ± 1.69	38.9 ± 1.69	16.3 *	16.1*	0.9	15.5 *
MQ4	60.6 ± 0.81	58.8 ± 0.98	56.2 ± 1.67	54.4 ± 1.67	45 ± 0.8	42.6 ± 0.98	55.7 ± 1.65	38.4 ± 1.65	15.6 *	16.2*	0.5	16 *

^1^ Tenderness, juiciness, flavor, and overall liking were scored by consumers on a 100 mm visual analogue scale ranging from 0 to 100. ^2^ Eating quality scores (MQ4) were calculated by weighting tenderness (0.4), juiciness (0.1), flavor (0.2), and overall liking (0.3) providing a MQ4 score between 0 and 100 [6]. ^3^ Data presented represents consumer sensory scores from 2080 individual untrained consumers. ^4,^* Within column indicates significant differences (*p* < 0.0001) between consumer sensory traits between striploins (*M. longissimus lumborum*) and outside flats (*M. biceps femoris*).
